# Monocyte Chemoattractant Protein-1 as a Biomarker in Acute Ischemic Stroke: A Prospective Pilot Study

**DOI:** 10.3390/jcm14103295

**Published:** 2025-05-09

**Authors:** Eleftheria Ztriva, Iraklis C. Moschonas, Alexandros Tselepis, Dimitrios Lambrou, Georgios Ntaios, Christos Savopoulos, Georgia Kaiafa

**Affiliations:** 1 First Propaedeutic Department of Internal Medicine, AHEPA University General Hospital of Thessaloniki, Aristotle University of Thessaloniki, 54636 Thessaloniki, Greece; elztriva@gmail.com (E.Z.); dnlambrou@gmail.com (D.L.); chrisavopoulos@gmail.com (C.S.); gdkaiafa@yahoo.gr (G.K.); 2 Atherothrombosis Research Centre/Laboratory of Biochemistry, Department of Chemistry, University of Ioannina, 45110 Ioannina, Greece; iraklismoschonas@hotmail.com (I.C.M.); atselep@uoi.gr (A.T.)

**Keywords:** acute ischemic stroke, monocyte chemoattractant protein-1, MCP-1, biomarker, NIHSS, mRS

## Abstract

**Background**: Monocyte chemotactic protein-1 (MCP-1) was implicated in the progression of atherosclerosis and is associated with elevated stroke risk. However, there is limited evidence regarding the MCP-1 role as an early biomarker for predicting the severity and outcomes of acute ischemic stroke (AIS). This prospective pilot case–control study aims to offer preliminary evidence into whether MCP-1 levels are elevated in AIS, whether they vary across different stroke subtypes, and their potential utility as a biomarker for assessing stroke severity and predicting outcomes. **Methods**: MCP-1 levels were quantified using ELISA in patients with AIS or transients ischemic attack (TIA) and healthy participants. Stroke severity was assessed with the NIHSS score and functional outcome with the mRS scale. **Results**: A total of 32 patients with AIS or TIA were compared to 13 healthy controls. MCP-1 levels were found to be 77% higher in stroke patients compared to healthy controls (*p* < 0.001). No significant differences in MCP-1 levels were observed between patients with AIS and those with TIA, nor among different stroke subtypes. A positive correlation was observed between MCP-1 levels and NIHSS changes from admission to discharge (b = 0.376, *p* < 0.05) and mRS scale at 6-month follow-up (b = 0.507, *p* < 0.05). **Conclusions**: This prospective pilot study provides preliminary evidence that MCP-1 levels are significantly elevated in AIS and are associated with NIHSS change during hospitalization and unfavorable outcome at 6-month follow-up. These findings indicate the potential of MCP-1 as an early biomarker for assessing disease severity and predicting outcomes in AIS.

## 1. Introduction

Stroke ranks as the third leading cause of death worldwide, with over 7 million deaths attributed to it [1], and it is the leading cause of long-term disability, placing a growing burden on global health systems [2,3]. Inflammatory mechanisms play a significant role in the pathophysiology of stroke. Cytokines and growth factors are key regulators of the inflammatory response and have been implicated in both the initiation and progression of atherosclerosis and vascular disease, including coronary artery disease, peripheral vascular disease, and stroke. Consequently, they may represent potential targets for the prevention and therapy of cardiovascular diseases [4].

A recent genome-wide association study conducted on Finnish healthy individuals uncovered several common genetic variants that affect the circulating levels of 41 cytokines and growth factors [5]. Their associations with stroke subtypes were evaluated in the MEGASTROKE genome-wide association study of 520,000 subjects [6]. A Mendelian randomization study has demonstrated that, among chemokines, a genetic predisposition to elevated circulating levels of monocyte chemotactic protein-1 (MCP-1) is associated with an increased risk of stroke, particularly large-artery atherosclerotic stroke and cardioembolic stroke [7].

MCP-1, also known as chemokine C-C motif ligand 2 (CCL2), is a chemokine that plays a critical role in attracting monocytes to sites of inflammation and it is primarily produced by inflammatory and endothelial cells [8]. MCP-1 is significantly involved in the initiation, development, and progression of atherosclerosis by facilitating the recruitment of monocytes into the forming atheromatous plaques [9,10]. In animal studies, the levels of MCP-1 and its receptor on monocytes, C-C chemokine receptor type 2 (CCR2), correlate closely with the severity of atherosclerosis and the degree of macrophage accumulation within the plaques [11].

A meta-analysis of six population-based cohort studies, which included 17,000 individuals without a history of stroke and followed them over 16 years, revealed that higher baseline levels of MCP-1 were linked to an increased risk of stroke, even after adjustment for traditional vascular risk factors. Combined with genetic and experimental data, these findings strengthen the evidence supporting MCP-1 as a causal risk factor for stroke, as well as a potential target for therapeutic intervention [12].

However, there are very limited data to prove that MCP-1 levels are elevated in acute phase of an ischemic stroke and that can be used as an early predictor to distinguish stroke subtype and subjective etiology or to estimate the severity and the disease outcome. This pilot preliminary study aims to assess whether MCP-1 levels are elevated during the acute phase of ischemic stroke and vary across stroke subtypes. Results support the design of a larger study aiming to examine in depth the role of MCP-1 for the prediction of early and long-term outcomes in patients after an ischemic stroke.

## 2. Materials and Methods

### 2.1. Study Design and Population

This is a case–control study conducted between January 2022 and July 2022. Patients with acute ischemic stroke (AIS) or transient ischemic attack (TIA) admitted to the Stroke Unit of AHEPA University Hospital in Thessaloniki, Greece, within 12 h of symptom onset or the last known well time were recruited along with healthy control subjects. Stroke pathophysiology was classified according to the TOAST criteria [13].

Inclusion criteria were as follows: age > 18 years old, AIS or TIA. Exclusion criteria were: intracranial hemorrhage, malignancy, central nervous or any other acute infection, and severe renal or hepatic disease.

Plasma levels of MCP-1 were measured by enzyme-linked immunosorbent assay (ELISA; Quantikine^®^ ELISA Human CCL2/MCP-1; R&D SYSTEMS, Minneapolis, MN, USA), following the manufacturer’s instructions. The reference interval of this assay is 31.3–2000 pg/mL, while the reference range of apparently healthy individuals is up to 436 pg/mL [14]. MCP-1 levels were compared between patients and healthy controls, and correlations between MCP-1 levels and comorbidities, as well as baseline laboratory values, were examined.

The National Institutes of Health Stroke Scale (NIHSS) was used for assessment of stroke severity [15,16] and modified Rankin Score (mRS) [17] was used for assessment of functional outcome. We also performed two additional analyses focusing on cardioembolic strokes vs. non-cardioembolic and on lacunar vs. non-lacunar strokes.

The outcomes of the study were as follows: NIHSS score at admission and discharge, mRS score at admission, discharge and at 6-month follow-up, NIHSS change from admission to discharge (δ-NIHSS), mRS change from prior to admission, from admission to discharge and from admission to 6-month follow-up, as well as in-hospital mortality and 12-month follow-up mortality.

### 2.2. Statistical Analysis

Quantitative data were summarized using mean values for parameters that are normally distributed and median values for parameters that are not normally distributed, while categorical data were described using frequency (count) and relative frequency (percentage). Comparisons between quantitative variables were performed using the independent *t*-test for normally distributed variables and the Kruskal–Wallis and Mann–Whitney tests for non-normally distributed data. Correlations between quantitative variables were analyzed using the Spearman correlation coefficient and linear regression analysis was conducted to estimate the regression coefficients. A *p*-value < 0.05 was considered statistically significant. Statistical analysis was conducted using SPSS (Statistical Package for the Social Sciences) version 23.

### 2.3. Ethical Review Board

The ethical review board of Aristotle University of Thessaloniki revised and approved the study protocol (No. 09/2022). All the study participants were treated according to the Helsinki declaration of Biomedical ethics and written informed consent was obtained from the patients or their caregivers.

## 3. Results

### 3.1. Baseline Characteristics

This study included 32 patients with AIS or TIA, with a median age of 80 years, of whom 13 were women (40.6%). Additionally, we recruited 13 healthy controls with a median age of 73 years (61.5% women). Patients with AIS were categorized into the following subtypes: nine large artery atherosclerotic, eight cardioembolic, four lacunar, and one of undetermined etiology. Table 1 summarizes the baseline characteristics of the recruited patients.

The median duration of hospitalization was 7 days. The median NIHSS at admission and at discharge was four and three, respectively. The median mRS before the AIS or TIA, at admission, at discharge, and at 6-month follow-up was two, four, four, and four respectively. Seventeen deaths were recorded at the 12-month follow-up, with eight deaths occurring during the in-hospital period. Among the causes of death, four were attributed to neurological deterioration, four to acute cardiovascular event, and nine to infections. Seven deaths (21.8%) were linked to confirmed (five cases) or suspected (two cases) SARS-CoV-2 infections.

### 3.2. MCP-1 Levels at Admission and Correlation with Baseline Characteristics

**Figure 1:**
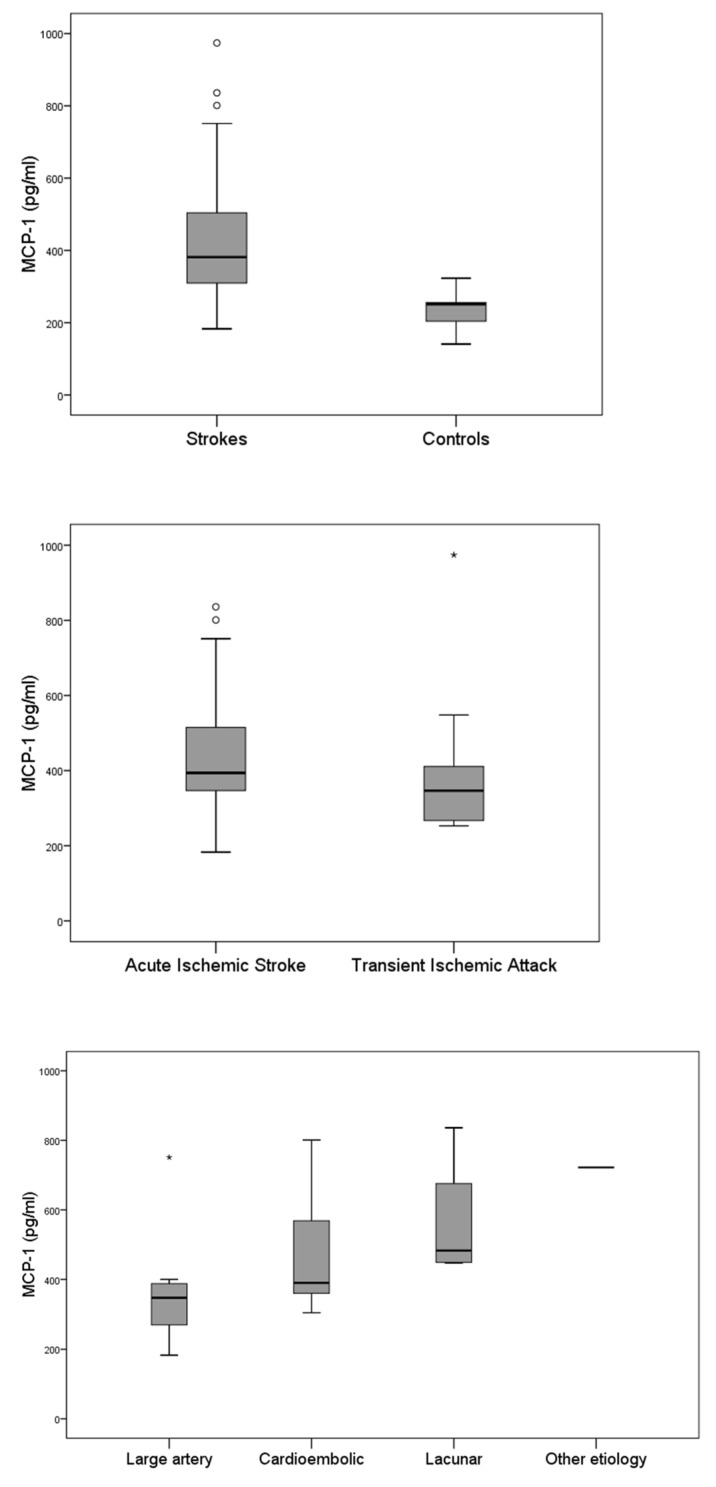
ΜCP-1 levels in the patient and healthy control groups (**upper panel**), acute ischemic strokes and TIA (**middle panel**), and according to the TOAST classification (**lower panel**). In the box plot, circles (○) represent mild outliers (1.5–3 × IQR beyond the quartiles), while asterisks (*) indicate extreme outliers (>3 × IQR).

At admission, the median MCP-1 levels were 381 pg/mL in patients compared to 251 pg/mL in healthy controls (*p* < 0.001). There was no difference in MCP-1 levels between patients with stroke and TIA (394 vs. 346 pg/mL, *p* = 0.309). Also, there was no difference in MCP-1 levels among different stroke types (Figure 1). More specifically, there was no difference in MCP-1 levels between cardioembolic and non-cardioembolic strokes (390 vs. 394 pg/mL, *p* = 0.707), but MCP-1 levels were higher in lacunar strokes compared to non-lacunar strokes, although this difference did not reach statistical significance (483 vs. 377 pg/mL, *p* = 0.061).

Νo correlation was observed between MCP-1 levels at admission and age, sex, the main comorbidities (arterial hypertension, diabetes mellitus, atrial fibrillation, dyslipidaemia, peripheral arterial disease, coronary disease, stroke history), the main laboratory values (plasma glucose, LDL-cholesterol, HDL-cholesterol, HBA1c), and arterial blood pressure at baseline (Table 2).

### 3.3. Correlation of MCP-1 Levels and Outcomes

**Figure 2:**
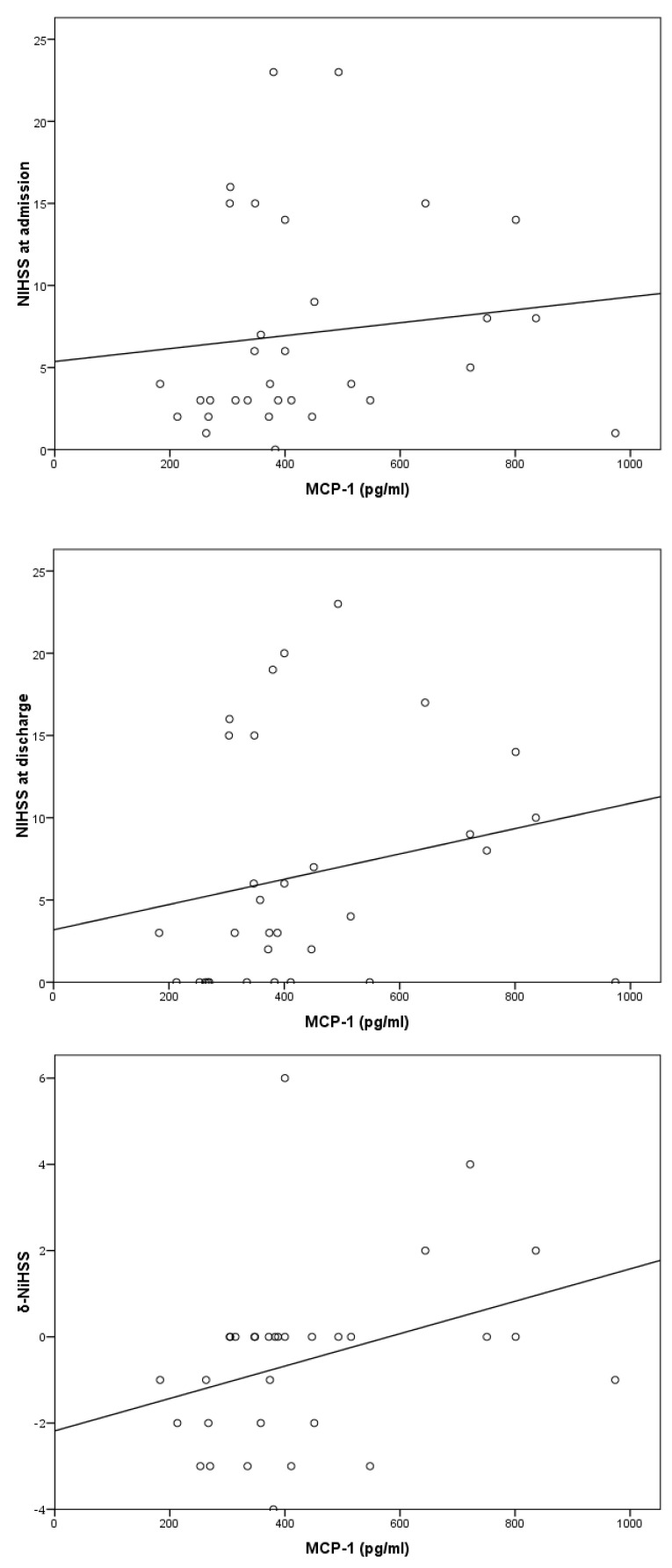
Correlation of MCP-1 to NIHSS at admission (**upper panel**), discharge (**middle panel**), and δ-NIHSS (**lower panel**).

A positive correlation was observed between the levels of MCP-1 and the difference in the NIHSS from admission to discharge from the hospital (δ-ΝIHSS) (*p* = 0.048) (Table 3 and Figure 2).

There was also a positive correlation between the levels of MCP-1 and the mRS score at 6-month follow-up (*p* = 0.03). However, there was no statistically significant association between MCP-1 levels and NIHSS or mRS score at admission and at discharge, nor with in-hospital mortality or death at 12 months (Table 3).

## 4. Discussion

In this prospective pilot study, we found that MCP-1 levels were higher in patients with AIS compared to healthy individuals. However, no significant differences in MCP-1 levels were identified between AIS and TIA or among different AIS subtypes classified by the TOAST criteria. A positive correlation was found between MCP-1 levels and the change in NIHSS scores from admission to discharge, as well as between MCP-1 levels and mRS score at 6-month follow-up. However, no significant correlations were observed between MCP-1 levels and NIHSS and mRS scores at admission and discharge, in-hospital mortality, or mortality at the 12-month follow-up.

The results of the present study demonstrate that the levels of the chemokine MCP-1 are elevated in the acute phase of ischemia compared to random samples from healthy controls. Our findings are consistent with evidence from various ischemia models, suggesting that elevated MCP-1 levels represent an immune response to ischemic injury and the coordinated recruitment of lymphocytes and monocytes to the site of damage shortly after the onset of ischemia [18].

Ischemic stroke is accompanied by an imbalance between the injured central nervous system and the immune system, resulting in a secondary condition of immunosuppression [19]. The ischemic core is characterized by the recruitment of neutrophils and monocytes/macrophages from the systemic circulation to the injured brain tissue [20]. As the blood–brain barrier loses its integrity, monocytes/macrophages, along with T-cells and NK cells, penetrate through the vascular endothelium to reach the damaged neural tissue. The initial purpose of this recruitment is to restore the immune response at site of local ischemia. However, it simultaneously increases the extent of local inflammation, contributing to extensive local brain damage and worse outcome in the ischemic stroke [19].

For this entire process, the damaged tissue expresses chemokine receptors on the surface of endothelial cells that interact with adhesion molecules on the surface of immune cells. Among chemokines, monocyte chemotactic protein-1 is primarily produced by inflammatory cells and endothelial cells and plays a critical role in attraction and recruitment of monocytes to sites of local brain inflammation [8]. However, activated monocytes can reduce the production of certain cytokines, leading to a decrease in MCP-1 secretion and, consequently, a reduction in the recruitment of additional monocytes to the site of inflammation [20,21]. Some authors suggest that the migration of monocytes out of the peripheral circulation decreases after the initial wave of mobilization, a decline that appears to be more pronounced in patients with TIA [21].

In our study, no difference was observed in the MCP-1 protein levels between patients with AIS and those with TIA, but this could be attributed to our limited sample size. According to the existing literature, monocytes accumulate in the injured tissue between days 3 and 6 post injury and infiltrate the damaged tissues within 72 h after the onset of the injury [20,22]. Therefore, during this period, a downregulation of MCP-1 could potentially occur, leading to lower MCP-1 plasma levels. It might be beneficial to design future studies with a larger sample size for each group, stroke vs. TIA, and reassess MCP-1 levels at different time points after the acute phase to prove this statement.

In our study, no significant difference was observed in MCP-1 levels when comparing among ischemic stroke subtypes. Given the distinct pathophysiological mechanisms, prognosis, and clinical features between cardioembolic and non-cardioembolic strokes [23,24], as well as lacunar and non-lacunar strokes [25], we examined these subgroups separately. However, no significant difference in MCP-1 levels was found. It is worth noting, though, that MCP-1 levels were higher in lacunar strokes compared to non-lacunar strokes, approaching statistical significance, although previous studies have suggested a correlation between elevated MCP-1 levels and an increased risk of large-artery atherosclerotic and cardioembolic strokes [7]. We hypothesize that, during the acute phase of cerebral ischemia, elevated MCP-1 levels are primarily reflective of the inflammatory response itself rather than being specific to the stroke etiology. Consequently, we may not expect differences in MCP-1 levels across different stroke subtypes. However, prospective cohort studies or meta-analyses of population-based follow-up studies may provide further knowledge whether MCP-1 levels are associated with specific ischemic stroke subtypes.

The levels of inflammatory markers and mediators in the blood following an acute ischemic stroke may vary and are associated with stroke outcomes [26]. It seems reasonable to hypothesize that an increase in MCP-1 levels would correlate with an advanced immune response and consequently greater neuronal damage. In our study, while we did not observe a difference in clinical severity during the acute phase of ischemic stroke in relation to MCP-1 levels, we did observe differences in clinical deterioration during the progression of ischemic stroke in the first days of hospitalization. That observation may indicate that immune response is ongoing during first days of stroke progression. Furthermore, elevated levels of MCP-1 during the acute phase of ischemic stroke have been associated with unfavorable outcomes at the 6-month follow-up. This finding underscores the potential utility of MCP-1 as an early biomarker for predicting long-term clinical outcomes as well.

No significant correlation was identified between the levels of MCP-1 and in-hospital or 12-month mortality. The reported mortality rate was higher than the mortality rate described typically in most acute stroke registries [27], but only one-quarter of the deaths were attributed to neurological deterioration. This was most probably attributed to the fact that the study was performed during the COVID-19 pandemic.

A recent study highlighted MCP-1 as a potential biomarker for identifying patients who could benefit from early antithrombotic therapy after an ischemic stroke [28]. However, other potential drugs targeting the immune response could also be applicable during the acute phase. Animal studies indicate that monocytes present at the site of ischemic injury play a role in promoting angiogenesis within the area of occlusion [21,29,30]. Considering that MCP-1 plays a crucial role in the migration and attachment of monocytes to brain injury sites, it may also contribute to angiogenesis. Therefore, targeting MCP-1 to modulate the immune response during the acute phase of ischemic stroke presents a promising therapeutic strategy. Such an approach could not only reduce neuronal damage by limiting excessive inflammation, but also promote therapeutic angiogenesis, thereby facilitating the recovery and repair of the damaged brain tissue.

Despite the insights provided by this prospective study, several limitations should be acknowledged when interpreting the results. First, the sample sizes of the stroke, TIA, and healthy cohorts, as well as the stroke subtypes, were relatively small, which limited the ability to match these cohorts for age, sex, and co-morbidities. Nevertheless, as this was a pilot study designed to explore the role of immunological biomarkers, such as MCP-1, in the pathophysiology of AIS and TIA, and to identify potential associations between MCP-1 levels and stroke outcomes, these limitations highlight the need for further prospective studies and randomized controlled trials with larger, more homogeneous cohorts. Such studies are essential to validate and extend these findings, ultimately facilitating the establishment of reliable, causal relationships. Additionally, MCP-1 levels were assessed only at a single time point during the acute phase of ischemic stroke. Repeated measurements at multiple time intervals can provide a more thorough understanding of the biomarker’s role in disease progression, especially across various stroke subtypes and TIA, offering a clearer picture of its dynamic changes over time.

In conclusion, this prospective pilot study provides evidence supporting the hypothesis that MCP-1 levels are elevated during the acute phase of ischemic stroke and these findings strengthen the need for further investigation through multicenter studies to evaluate the potential utility of MCP-1 as a biomarker in AIS. MCP-1 could serve as an early biological marker for assessing disease severity and predicting disease progression. Monitoring plasma MCP-1 levels may become an essential component of the management protocol for patients during the acute phase of ischemic stroke. Given the distinct pathophysiology underlying each stroke subtype, further investigation into the role of immunological biomarkers such as MCP-1 across different stroke subtypes is critical, as they may emerge as potential therapeutic targets. Further research into MCP-1 may be proven useful in developing therapeutic agents aiming at immune modulation. Considering the established pathways of the immune response in ischemic stroke, targeting the inhibition of the pro-inflammatory immune response could serve as a therapeutic approach to prevent secondary damage while maintaining the anti-inflammatory response and its immunoprotective effects.

## Figures and Tables

**Table 1:** Baseline characteristics of patients with acute ischemic stroke or TIA at admission.

Variables	Acute Ischemic Stroke	Transient Ischemic Attack
	*n* = 22	*n* = 10
Age, years	79 (12)	84 (14)
Female sex	10 (45.5)	3 (30)
Arterial hypertension	17 (77.3)	8 (80)
Diabetes mellitus	7 (31.8)	2 (20)
Dyslipidemia	10 (45.5)	0 (0)
Atrial Fibrillation	9 (40.9)	5 (50)
Coronary artery disease	8 (36.4)	4 (40)
Peripheral artery disease	4 (18.2)	0 (0)
Previous stroke	7 (31.8)	6 (60)
Current smoking	7 (31.8)	3 (30)
MCP-1, pg/dL	394 (210)	346 (179)
LDL-cholesterol, mg/dL	85 (59)	97 (62)
HDL-cholesterol, mg/dL	39 (24)	35 (15)
Glucose, mg/dL	116 (65)	113 (51)
HBA1c, %	6.1 (1.1)	5.9 (0.7)
Systolic blood pressure, mmHg	160 (49)	145 (31)
Diastolic blood pressure, mmHg	80 (12)	73 (18)
NIHSS at admission	7 (11)	3 (2)
mRS before admission	1 (2)	2 (3)
mRS at admission	4 (2)	4 (3)

Numbers represent median values and interquartile range (IQR) for continuous variables, and absolute counts and proportions for categorical variables. MCP-1: Monocyte chemotactic protein-1; LDL-cholesterol: Low-density lipoprotein–cholesterol; HDL-cholesterol: High-density lipoprotein–cholesterol; HbA1c: Hemoglobin A1c; NIHSS: National Institutes of Health Stroke Scale; mRS: modified Rankin Scale.

**Table 2:** Correlation of MCP-1 levels with baseline characteristics.

Variables	Regression Coefficient	*p* Value
Age	2.15	0.574
Sex	30.73	0.667
Arterial hypertension	103.76	0.216
Diabetes mellitus	−110.31	0.150
Dyslipidemia	32.32	0.151
Atrial fibrilation	−14.08	0.152
Peripheral arterial disease	47.36	0.153
Coronary artery disease	−112.90	0.154
Stroke history	−10.34	0.155
Current smoking	−81.27	0.156
Alcohol	66.14	0.157
LDL-cholesterol	1.21	0.158
HDL-cholesterol	1.10	0.159
HbA1c	−7.72	0.160
Systolic blood pressure	0.46	0.161
Diastolic blood pressure	0.73	0.162
Glucose	1.29	0.164

LDL-cholesterol: Low-density lipoprotein-cholesterol; HDL-cholesterol: High-density lipoprotein-cholesterol; HbA1c: Hemoglobin A1c.

**Table 3:** Correlation of MCP-1 levels with outcomes.

Outcomes	Regression Coefficient	*p* Value
Days of hospital stay	0.280	0.725
NIHSS at admission	0.394	0.513
NIHSS at discharge	0.769	0.248
δ-NIHSS	0.376	0.048
mRS before admission	−0.084	0.455
mRS at admission	0.067	0.623
mRS at discharge	0.214	0.267
mRS at 6 months	0.507	0.030
In-hospital death	0.173	0.393
Death at 12 months	0.378	0.074

NIHSS: National Institutes of Health Stroke Scale; δ-NIHSS: NIHSS change from admission to discharge; mRS: modified Rankin Scale.

## Data Availability

The data presented in this study are not available due to privacy and ethical restrictions.

## Abbreviations

The following abbreviations are used in this manuscript:

δ-NIHSS	NIHSS change from admission to discharge
Adm	Admission
AF	Atrial fibrillation
AIS	Acute ischemic stroke
CCL2	Chemokine (C-C motif) ligand 2
CCR2	C-C chemokine receptor type 2
CN	Coronary disease
DaysHosp	Days of hospital stay
Diff	Difference
Dis	Discharge
DM	Diabetes mellitus
HbA1c	Hemoglobin A1c
HDL-cholesterol	High-density lipoprotein–cholesterol
LDL-cholesterol	Low-density lipoprotein–cholesterol
MCP-1	Monocyte chemotactic protein-1
mRS	Modified Rankin score
NIHSS	National Institutes of Health Stroke Scale
PVD	Peripheral vascular disease
Pre	Prior to admission
SPSS	Statistical package for the social sciences
TIA	Transient ischemic attack
TOAST	Trial of Org 10,172 in acute stroke treatment
